# ARG-walker: inference of individual specific strengths of meiotic recombination hotspots by population genomics analysis

**DOI:** 10.1186/1471-2164-16-S12-S1

**Published:** 2015-12-09

**Authors:** Hao Chen, Peng Yang, Jing Guo, Chee Keong Kwoh, Teresa M Przytycka, Jie Zheng

**Affiliations:** 1 Biomedical Informatics Graduate Lab, School of Computer Engineering, Nanyang Technological University, 50 Nanyang Avenue, Singapore 639798, Singapore; 2 Singapore Immunology Network (SIgN), A*STAR, Biopolis, Singapore 138648, Singapore; 3 Institute for Infocomm Research (I2R), A*STAR (Agency for Science, Technology, and Research), 1 Fusionopolis, Singapore 138632, Singapore; 4 Computational Biology Branch, National Center for Biotechnology Information (NCBI), National Library of Medicine (NLM), National Institutes of Health (NIH), 8600 Rockville Pike, Bethesda, Maryland 20894, USA; 5 Genome Institute of Singapore, A*STAR, Biopolis, Singapore 138672, Singapore

**Keywords:** Meiotic recombination hotspot, individual recombination strength, ancestral recombination graph (ARG), genome-wide association study (GWAS), major histocompatibility complex (MHC), random walks on graphs

## Abstract

**Background:**

Meiotic recombination hotspots play important roles in various aspects of genomics, but the underlying mechanisms for regulating the locations and strengths of recombination hotspots are not yet fully revealed. Most existing algorithms for estimating recombination rates from sequence polymorphism data can only output average recombination rates of a population, although there is evidence for the heterogeneity in recombination rates among individuals. For genome-wide association studies (GWAS) of recombination hotspots, an efficient algorithm that estimates the individualized strengths of recombination hotspots is highly desirable.

**Results:**

In this work, we propose a novel graph mining algorithm named ARG-walker, based on random walks on ancestral recombination graphs (ARG), to estimate individual-specific recombination hotspot strengths. Extensive simulations demonstrate that ARG-walker is able to distinguish the hot allele of a recombination hotspot from the cold allele. Integrated with output of ARG-walker, we performed GWAS on the phased haplotype data of the 22 autosome chromosomes of the HapMap Asian population samples of Chinese and Japanese (JPT+CHB). Significant *cis-*regulatory signals have been detected, which is corroborated by the enrichment of the well-known 13-mer motif CCNCCNTNNCCNC of *PRDM9 *protein. Moreover, two new DNA motifs have been identified in the flanking regions of the significantly associated SNPs (single nucleotide polymorphisms), which are likely to be new *cis-*regulatory elements of meiotic recombination hotspots of the human genome.

**Conclusions:**

Our results on both simulated and real data suggest that ARG-walker is a promising new method for estimating the individual recombination variations. In the future, it could be used to uncover the mechanisms of recombination regulation and human diseases related with recombination hotspots.

## Background

Meiotic recombination is a crucial step in the reproduction of many species. The reciprocal exchange of genetic material between homologous chromosomes during the meiosis (i.e. meiotic recombination) is an important evolutionary force for increasing genetic diversity and also essential for proper chromosome segregation. Knowledge about recombination is important for understanding the linkage disequilibrium (LD) structure of the genome [1], phenotypic diversity and evolution in a population [2], and a variety of genetic diseases [3]. Recombination events do not occur randomly along the chromosomal DNA, but would rather cluster on short chromosomal intervals, typically 1-2 kb long, named recombination hotspots [4]. Recent development in the construction of genome-scale, high-resolution recombination map and new biological techniques for analysing this cellular progress have provided a comprehensive view of the distribution of recombination hotspots as well as insights into the regulatory systems that control the recombination landscape. In particular, the *PRDM9 *protein was found to be an important *trans*-regulator controlling the activity of recombination hotspots through binding to a 13-mer DNA motif (CCNCCNTNNCCNC) in the human genome [5-8]. However, the binding motif of *PRDM9 *does not cover all the human hotspots [7], and there still exists a substantial gap in our understanding of the regulatory system. Moreover, open questions and challenges remain, such as the recombination variation in association with gender and age, epigenetic regulators of meiotic recombination, the roles of recombination hotspots in human diseases, etc. Thus finding other *trans*- and *cis*- regulators that can also regulate recombination hotspots is highly desired.

Most existing methods for the statistical analysis of recombination mainly rely on the estimation of average recombination rate from a population, such as coalescent based analysis of linkage disequilibrium (LD) [4,9]. Software tools have been developed to help researchers identify recombination hotspots by LD analysis, such as LDsplit [10-13] which has been applied to disease study [14]. Yet the power of individual recombination events tends to be overlooked by most approaches despite the accumulating evidence that recombination frequencies differ significantly between ethnic groups, genders, and also among individuals [15-17]. Although some methods including pedigree analysis [17,18] and sperm typing [19] can handle sex-specific and individual recombination analysis, they are limited by technical factors such as the high cost, short regions, or low resolution. Therefore, algorithms that can infer individual-specific recombination rates both on a large scale and with high resolution would be very useful for genomic studies of recombination hotspots.

Ancestral recombination graph (ARG) is a topological structure that captures the genealogical history of individuals, including historical mutations, recombination events and merging, back to a common ancestor. Thus ARG is indispensable for study of the recombination events in various evolutionary scenarios. Despite the computational complexity of the ARG inference, in recent years, there emerged several methods that have largely solved this problem. For example the algorithm of IRiS [20] can detect past recombination events based on a graph reconstruction algorithm [21] followed by integrating these recombination events into a subARG; ACG [22] estimates the full likelihood of the ARG using a Bayesian Markov chain Monte Carlo (MCMC) procedure; ARGweaver [23] can infer ARGs from genome-wide data based on hidden Markov models (HMM).

In this paper, we propose a graph mining method, namely ARG-walker, to infer the different strengths of a recombination hotspot among individuals in a sample. Given a set of extant haplotypes, we first adapt the IRiS algorithm to detect the recombination events and integrate them to construct the ARG; then a random walk method is applied on the ARG to estimate the individual-specific recombination propensities; as such, ARG-walker can translate SNP sequences to a recombination profile, i.e. a vector of floating numbers each representing the corresponding individual strength of a recombination hotspot. This method can be used to exploit the power of the variation in individual recombination frequency, to shed light on the regulatory system of recombination hotspots. Our extensive simulation tests demonstrated the statistical power of ARG-walker in detecting phenotypic variations of recombination rate. Then, applying ARG-walker on the HapMap phased SNP data for GWAS of recombination hotspots, we detected strong association signals of *cis*-regulation, corroborated by the enrichment of the aforementioned 13-mer *PRDM9 *binding motif CCNCCNTNNCCNC in proximal regions of SNPs identified by our GWAS. Through further analysis of the flanking DNA sequences of associated SNPs, we found two new motifs, AAAATANA and CNGCCTCC, which could be potential *cis-*regulators of the meiosis recombination hotspots in the human genome. Moreover, by screening the GWAS results on MHC (major histocompatibility complex) region in human chromosome 6, we detected two significantly associated SNPs, *rs576205 *and *rs2061915*. The two SNPs are located in the coding regions of *KSR2 *and *ZNF708 *protein respectively, both of which have been reported to have regulatory roles in T-cell activation. It demonstrates the potential of ARG-walker for detecting *trans-*factors of meiotic recombination hotspots, and for study of recombination hotspots important in the human immune system.

## Methods

### Experimental data

The input data of ARG-walker consist of SNP data generated by simulations or collected by the HapMap project. Our simulation data was generated by a Python script which we developed using Python 2.6 and based on simuPOP (version 1.0.3) [24] which is an open source framework for forward simulation of population genetics. To evaluate the performance of ARG-walker under different situations, we set multiple groups of parameters in simuPOP according to different scenarios. Each simulation data set consists of haplotypes of 90 individuals, each individual has two homologous haplotypes of about 100 SNPs (the number of SNPs was randomly generated to be near 100) spanning 200 kb, and each SNP has two alleles, denoted as 0 and 1 (Additional File 1). For testing ARG-walker on the real data, we used the SNPs in the 22 human autosome chromosomes of HapMap Phase 3 data and recombination hotspots therein detected by LDhat [25]. We first extracted the haplotypes of 22 autosome chromosomes of JPT+CHB (Japanese and Chinese) population of the HapMap Phase 3 dataset. The length of each haplotype sequence was set to 100 SNPs and overlapped haplotypes were filtered, also the pre-processing required for running the IRiS software was applied to filter SNPs with minor allele frequency (MAF) less than 0.01, and non-tag SNPs were removed from the sequences. In this way we obtained 8,721 haplotype sequences with hotspots in the middle. Then, the haplotypes were fed to ARG-walker to estimate the individual-specific strengths of the recombination hotspot located inside the 100-SNP window. For each hotspot, a profile of the estimated recombination strengths will be output. For the genotype data, we applied the FastTagger [26] tool to select all tag SNPs with MAF higher than 0.3 from the HapMap Phase 3 data of JPT+CHB population. The minimal *r*^2 ^was set to 0.9. As such we obtained 179,671 tag SNPs. The Python script for generating the simulation data and Perl scripts for extracting and pre-processing of these SNP data are available in Additional File 1.

### Description of ARG-walker

**Figure 1:**
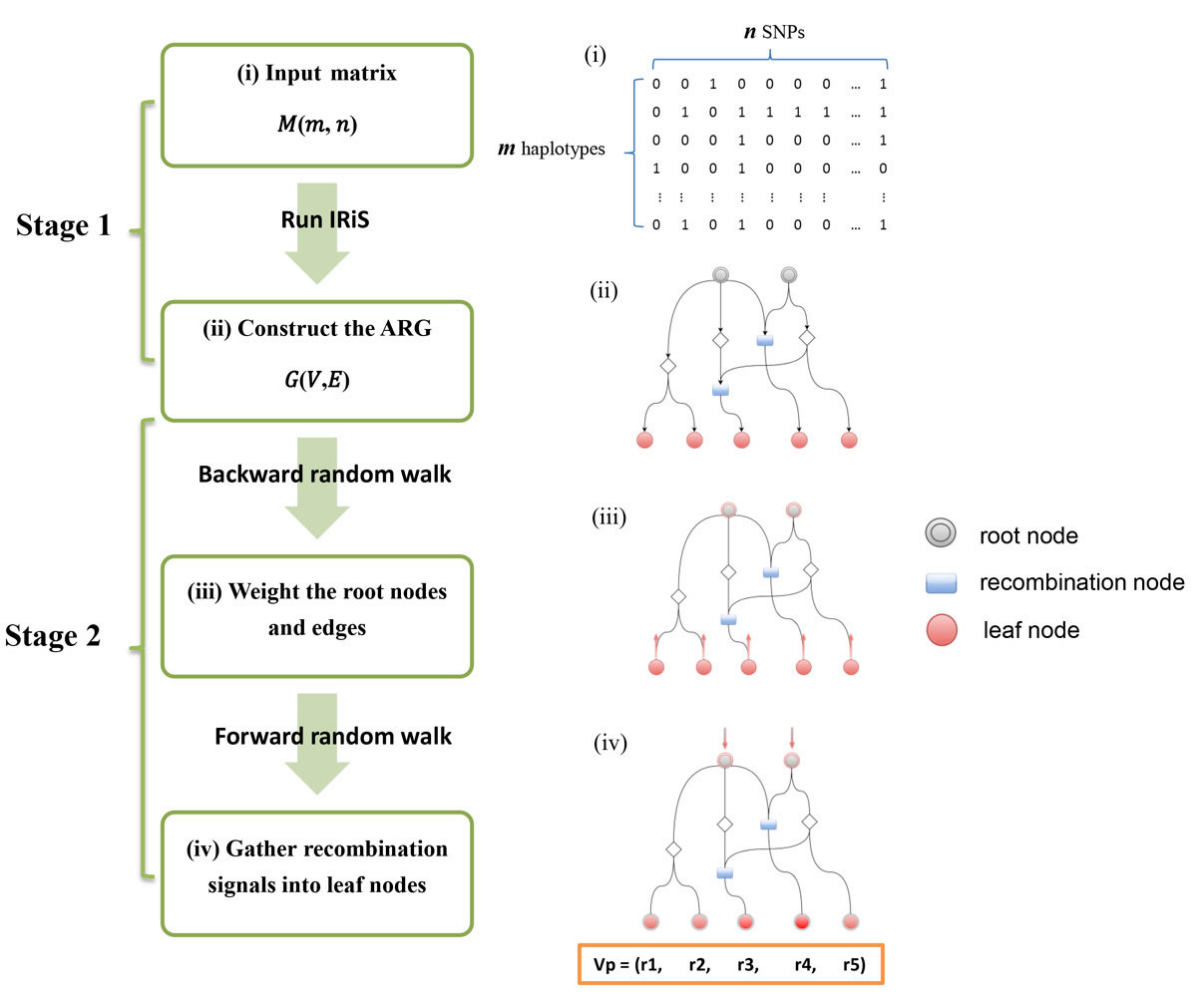
**The pipeline diagram of ARG-walker algorithm**. ARG-walker consists of two stages. In Stage 1, from an input sample of haplotypes, the IRiS program is used to reconstruct the ancestral recombination graph (ARG), and the nodes are classified into three types as illustrated with different shapes and colours. In Stage 2, information flows indicating signals of recombination are first gathered to the root nodes through backward random walk, then propagated downwards through forward random walk and in the end gathered by the leaf nodes. The red arrows in Step (iii) and Step (iv) illustrate the start of the random walks.

The main idea of ARG-walker is to estimate, through analysis of ARG topology, the frequencies of historical recombinations, which are then used to approximate the recombination propensities of extant individual chromosomes. First, to reconstruct the ARG, we use the IRiS algorithm which is able to infer historical recombination events from a set of extant haplotypes and integrate these events into an ARG [27]. Then, we apply a random walk algorithm to mining the ARG in order to estimate the recombination strengths of individuals corresponding to the input haplotypes. With random walks on the ARG, we assign positive weights indicating signals of recombination to the root nodes, and then like raindrop collection, the information flow runs from top to bottom of the ARG. If one extant haplotype has more ancestral recombination events in the history, then more information flows, flowing downward along the paths in the ARG, will be gathered at the downstream ARG leaf nodes corresponding to the haplotypes in the end. As such, individuals with different recombination histories can be distinguished by the amounts of information they collected from the random walks, which represent their strengths of recombination hotspot. The algorithm of ARG-walker, which consists of two stages, is illustrated in Figure 1, and described as follows.

### Stage 1: ARG construction and node classification

The input of our method consists of a sample of extant human haplotypes. The software of IRiS is employed to reconstruct the ARG from the input haplotype sample. IRiS first cuts the haplotypes into segments, from which phylogenetic forests can be inferred to explain the segmentation. From the segments and forests, compatible sub-networks are constructed using an algorithm called DSR (dominant, subdominant or recombinant), which is a greedy algorithm that attempts to minimize the number of recombinations needed to explain the given data. These sub-networks are merged to construct the ARG represented as a directed graph *G*(*V*, *E*). Readers interested in details of the algorithm behind IRiS are referred to [20,21]. For each node *ν *∈ *V*, three types of degrees are calculated, i.e. indegree deg_-_(*ν*), outdegree deg_+-_(*ν*) and degree deg(*ν*) = deg_-_(*ν*) + deg_+_(*ν*). With the information of degrees, the nodes are classified into three types: (1) the root nodes in set *R*_*v *_with deg_-_(*ν*) = 0 and deg_+_(*ν*) > 0, (2) the recombination nodes in set *Recom_v _*with deg_-_(*ν*) ≥ 2 and deg_+_(*ν*) > 0, and (3) the leaf nodes with deg_-_(*ν*) > 0 and deg_+_(*ν*) = 0. Here each node is represented by an integer, which is an index in the vertex set *V*. Note that the ARG constructed by IRiS could contain more than one root nodes because the reconstructed graph is only partial ARG (or subARG), which may contain only a subset of nodes and edges of the true ARG. For conciseness, however, we will hereafter still refer to the output of IRiS as ARG, rather than subARG.

### Stage 2: ARG mining using backward-forward random walks

After the nodes are classified, a backward random walk from the leaf nodes to the root nodes is conducted to assign weights on the edges which are stored into an edge-weight matrix *W*_*n*×*n*_, where *n *is the number of nodes in the ARG. Initially, each entry of the matrix *W *is set to 0. Then, the matrix entries are iteratively updated according to the topological structure of the ARG, following the rule that $W_{ij}=1+\sum_{k\neq i,k\in V}W_{jk}$ if there is a directed edge from node *j *to node *i *in the ARG. In other words, the weight of the edge from node *j *to node *i *is set to be the number of all descendants of node *j *plus one. If there is no edge from node *j *to node *i*, the corresponding entry in the matrix *W *will be 0. Due to its Y-shape structure in the ARG, a recombination node will be double-counted as two descendants, and as a result, edges with more recombination nodes as descendants can collect larger weights through the backward walk. Later the edge weights will be passed downward all the way to the leave nodes to reflect the inherited propensity of recombination. To apply the forward random walk on the ARG, we transform the edge-weight matrix *W*_*n*×*n *_into the transition probability matrix *T*_*n*×*n *_by normalization, i.e. $T_{ij}=\frac{W_{ij}}{\sum_{k\in V}W_{ik}}$. Through forward random walk the signals of recombination will be passed from the root nodes layer by layer towards the leaf nodes. For a coalescent node, the signal will be split to two children proportional to the transition probabilities, whereas for a recombination node in the next generation, the signals from two parental nodes will be combined. The procedure is executed by iteratively updating two vectors, each consisting of *n *entries corresponding to the ARG nodes. The first vector, denoted by *I*, contains the amounts of signals flowed to ARG nodes at the end of a step, which will be passed to their children in the next step. The second vector, denoted by *V_L_*, records the amounts of signals that the leaf nodes will gather at the end of the random walk. At the beginning, vector *I *is initialized by setting each entry corresponding to a root note equal to the sum of weights of its outgoing edges, and setting other entries to 0, i.e. $I\left[i\right]=\{\begin{array}{ll} \sum_{k\in V}W_{ik}, & \text{if }i\in R_{v}\\ 0, & \text{otherwise} \end{array}$ for *i *= 1,2,...,*n*. Vector *V_L _*consists of all 0s initially. For each iteration of the forward random walk, the following two operations are carried out to update the two vectors: (1) *I *= *I *× *T*, and (2) *V_L _*= *V_L _*+ *I*. Note that an entry of the transition probability matrix, say *T_uv _*, contains a positive value if there is a directed edge from node *u *to node *v*, and is equal to 0 otherwise. Thus, by the first operation of *I *= *I *× *T*, the signal in a node *i *will all be passed to its child nodes in proportion to the transition probabilities. This iterative procedure is repeated until every entry of vector *I *is equal to 0, which means every node has no more information to pass on. After the repeated updates, the signals are collected in vector *V_L _*by the second operation above. But we will set to 0 all the entries in *V_L _*that do not correspond to the leaf nodes, so that *V_L _*only contains signals of recombination of the extant haplotypes. Moreover, the recombination probability of each individual leaf node is estimated by normalization of *V_L_*, i.e. $V_{p}\left[i\right]=\frac{V_{L}\left[i\right]}{\sum_{j\in L}V_{L}\left[j\right]}$, where *L *is the set of indices of leaf nodes in the vertex set of ARG, and *i *∈ *L*. Thus the final result of ARG-walker is a vector *V_L _*which contains the amount of information flow gathered by each individual haplotype corresponding to a leaf node in the ARG. This information flow is used to represent the strength of recombination of each individual chromosome. In addition, vector *V_p _*is also output to represent the recombination probability of each individual chromosome. ARG-walker was developed in Perl (v5.16.3) and the source code is available in Additional File 2.

## Filtering hotspots without variation in recombination strength

In the simulation, we adapted the Hartigan's dip test [28] to test the unimodality of the distribution of estimated recombination strengths. This is for the case when there are only small variations of recombination strength in a population, e.g. the majority of haplotypes have cold (or hot) alleles, or it is hard to divide the population into two phenotypic groups. In our method, we assumed that when there are both hot and cold alleles of a recombination hotspot, the distribution of recombination strengths given by ARG-walker should be more likely to be bimodal. In the dip test for these two situations, we found significant difference of p values between these two groups. With a threshold of 2.473 of the dip −log_10_(p) value, we can filter out 88% samples with non-variant recombination strengths, while keeping 87.8% samples of two-allele recombination strengths. Applying this strategy in our GWAS analysis, we selected 5,200 recombination hotspots (each with a profile of individual strengths) as phenotypes.

## Combination of recombination phenotype by chromosome

Besides individual hotspot phenotype analysis, we also merged recombination strengths of hotspots on the same chromosome into one collective chromosome-wise phenotype. First, we did standardization for each hotspot using formula (*x *- *mean*)/*SD*, and then we combined the standardized hotspot phenotypes by chromosomes, i.e. the mean value of all the standardized hotspot phenotypes on the same chromosome was calculated to represent the chromosomal recombination strength of each individual. Finally we got 22 combined chromosomal recombination phenotypes. Each of this newly generated phenotype was then mapped to the genotypes for GWAS analysis.

## Results and discussion

### Simulation study

**Figure 2:**
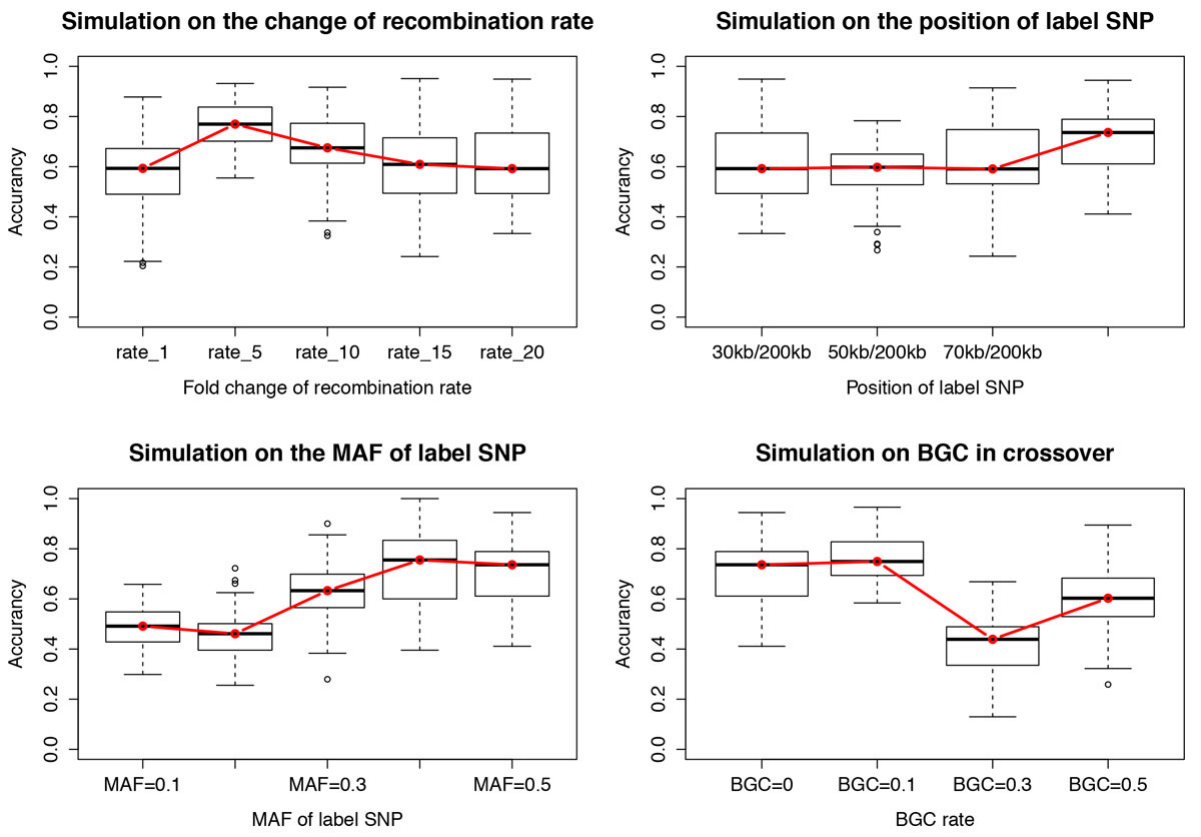
**The accuracy of prediction on simulation data with 4 main parameters**. The changes in accuracy of ARG-walker were tested with regard to four main parameters in our simulation study, i.e. average recombination rate, the position of the causal SNP, MAF of the causal SNP and the biased gene conversion (BGC) rate. 50 sample files were generated for each simulation. For each test, the distribution of accuracies of ARG-walker was plotted as a boxplot, where the red line connects the median values of accuracy.

The evolution of meiotic recombination hotspots is notoriously dynamic and complicated, partly due to processes such as biased gene conversion (BGC). To slightly simplify our simulation (without making the synthetic data unrealistic), we focused on the common scenario of two-allele variation of recombination, i.e. hot versus cold alleles in a population. Currently large amounts of evidence from both sperm typing and genetic analyses suggest that the general patterns of recombination in the human genomes are highly unstable throughout the genome [4,9,29]. Hence, we tested different sets of parameters to simulate different recombination scenarios, of which key parameters include the recombination rate, the position of labelled SNP, MAF of the labelled SNP and the BGC rate. For each set of parameters, we generated 50 samples, and fed each sample to ARG-walker. Then, the accuracy was calculated and presented with a boxplot. We compared the performance of ARG-walker under different situations by the boxplot of accuracy with the median accuracy connected with a red line in Figure 2. First, we tested the change of recombination rates including the crossover rates of the hot individuals and the cold individuals. The ratio of these two crossover rates was set to 1 which means equal rate, 5, 10, 15 and 20. The result shows that our method has better performance with the increase of hot/cold ratio from 0 to 5, while the accuracy is slightly decreased and seems to become stable when the ratio is higher than 15. This suggests that some recombination events are not detected by ARG-walker, probably due to the loss of traces on the patterns of linkage disequilibrium when there is a high frequency of recombination. Then we analysed the effect of the position of the SNP controlling crossover rate, from the centre to the far ends of the window. The accuracy of our method does not fluctuate much, but with a tendency that the accuracy gets higher when the causal SNP is closer to the centre. Another important factor we tested is the MAF of the causal SNP. When the MAF is low, say between 0.1 and 0.2, the performance of ARG-walker is poor, with accuracy less than 0.5. A lower MAF means a lack of homogeneity of phenotype in the sample, which shall be filtered out before using ARG-walker. When the MAF is bigger than 0.3, however, ARG-walker is able to achieve a higher accuracy. Another key parameter is the BGC rate along with a crossover. BGC was suggested to be in favour of hotspot-disrupting alleles thus it has crucial influence on the recombination hotspots and their evolution [30]. Our result shows that ARG-walker can be affected to some degree by BGC but it still achieved good performance when the BGC rate is lower than 0.3 (Figure 2, bottom-right boxplot).

**Figure 3:**
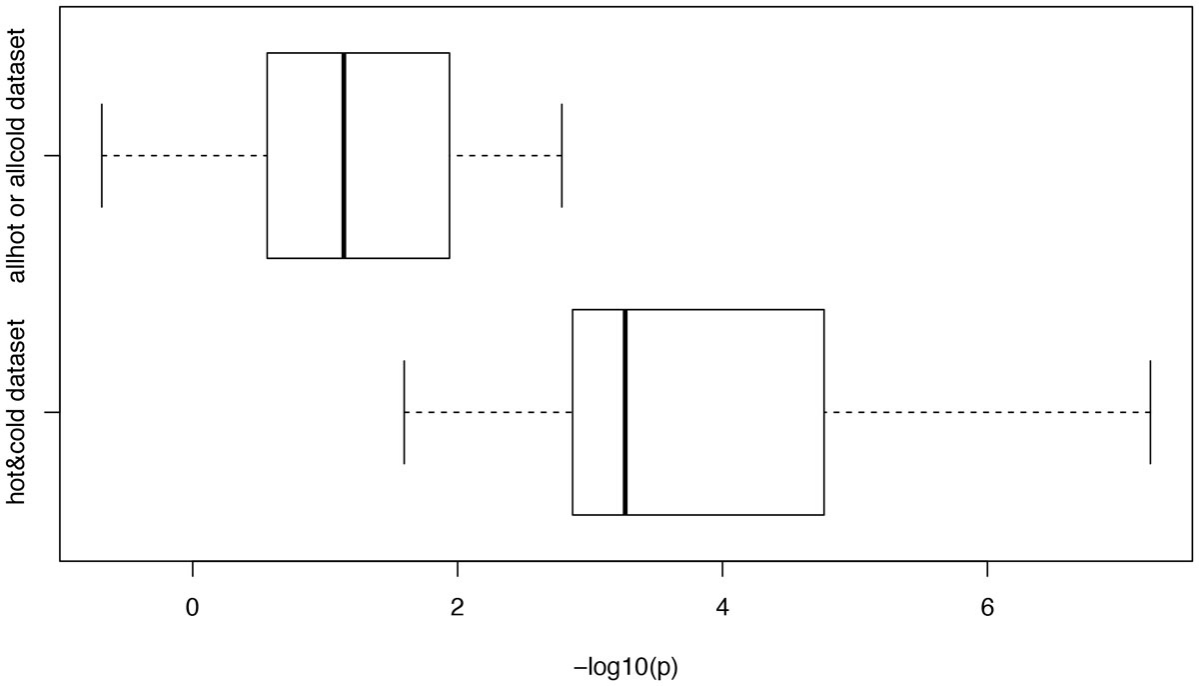
**Boxplot of log transformed dip p value of all-hot or all-cold cases (on the top) and two-allele hot-cold cases (at the bottom)**.

**Figure 4:**
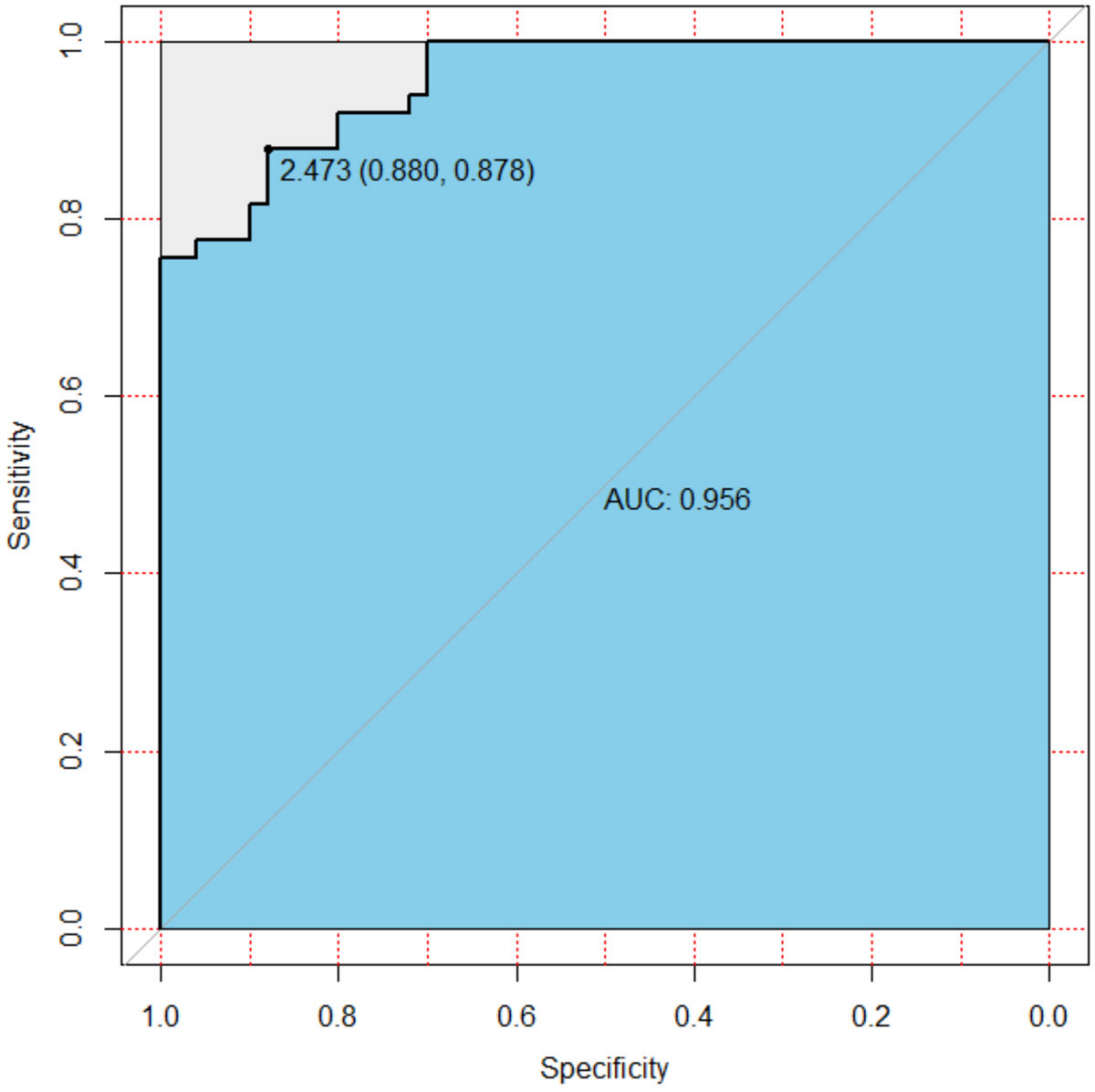
**ROC curve of dip test for differentiating all hot/all cold cases from hot-cold cases**.

In addition to these tests of parameters, we also investigated two special cases, i.e. all-hot and all-cold. In these two cases, there is no difference in recombination strengths among individuals. Hartigan's dip test was used to test if our ARG-walker can identify these two special cases (see details in Methods section). In contrast to two-allele cases, here the test shows a significant difference on dip p-values, as shown in the boxplot in Figure 3. Moreover, the ROC curve in Figure 4 shows a very high AUC of 0.956 indicating that a threshold of 2.473 can dramatically differentiate these two scenarios with a specificity of 0.88 and a sensitivity of 0.878.

In summary, with relatively higher differences in recombination rates, an MAF of 0.3 or higher at the causal SNP, and BGC rate less than 0.3, our ARG-walker has a reasonably good performance of prediction, with the average accuracy about 0.64 (Table 1). In addition to the hot-cold variation, ARG-walker can also identify the all-hot and all-cold cases, which is important for GWAS of recombination phenotypes later.

**Table 1:** Average performance of ARG-walker on simulation data

Condition	Sensitivity (%)	Specificity (%)	Accuracy (%)
Variant recombination rate	71.48	57.13	64.16
Variant position of causal SNP	71.72	54.64	63.08
Variant MAF of causal SNP	65.75	45.39	60.35
Variant BGC rate	70.49	53.06	61.55
Normal	81.73	59.37	70.19

### Genome-wide association study of recombination hotspots

**Figure 5:**
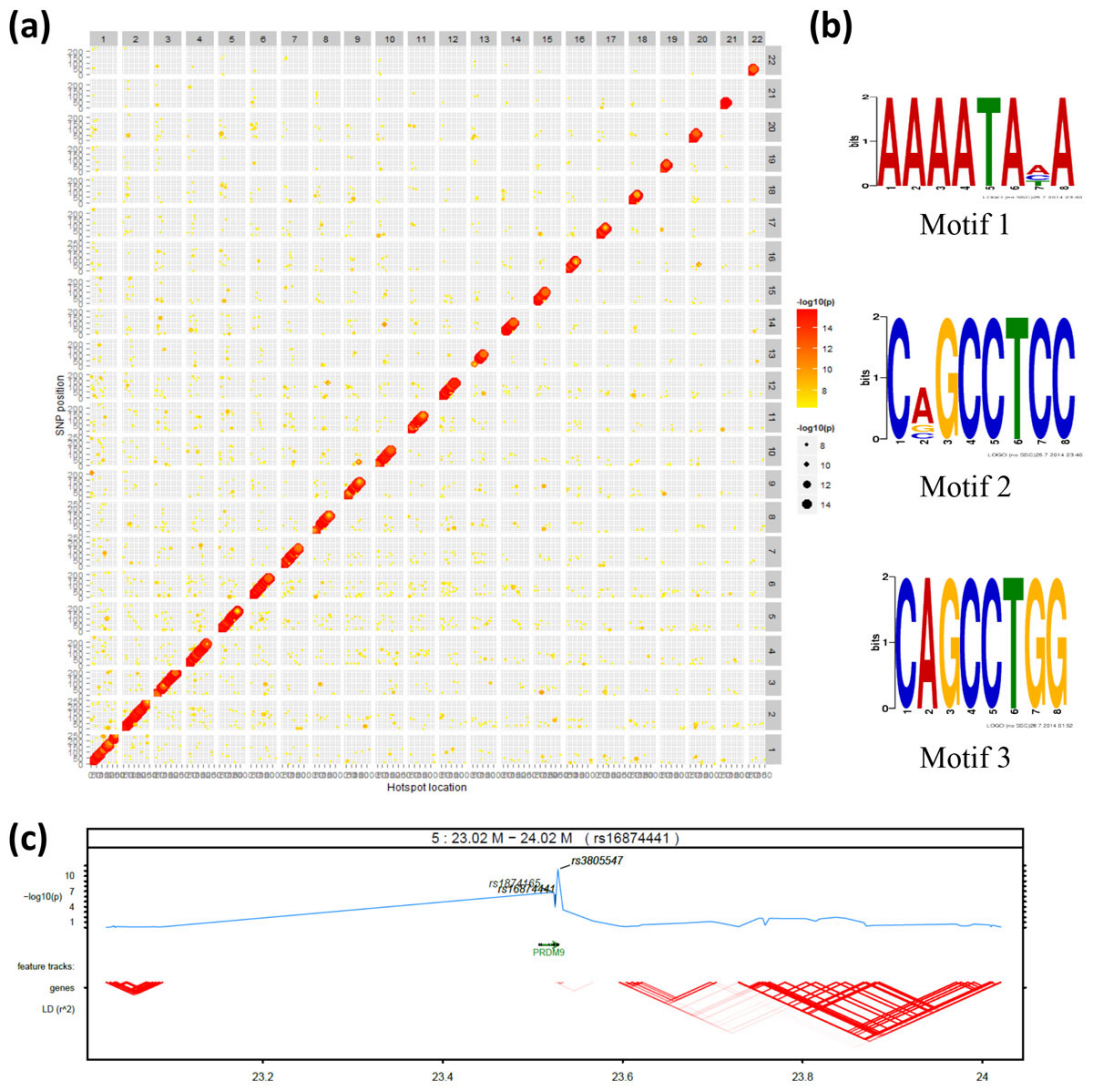
**Grid dot plot of genome-wide association study of recombination hotspots, DNA motifs enriched in flanking sequences of associated SNPs and regional plot of SNPs inside *Prdm9 *gene**. (a) Dot plot of the genome-wide association between meiotic recombination hotspots and tag SNPs with gradient colour intensity and size representing the strength of association. (b) Three top ranking motifs detected by DREME from the 1 kb DNA sequences flanking significant SNPs predicted by ARG-walker and GWAS. (c) Regional plot of the SNPs located inside *Prdm9 *gene with log transformed p-value of association, feature track, genes and LD.

A major motivation of estimating the individual-specific strengths of recombination hotspots is to perform genome-wide association study (GWAS) to identify *trans- *and *cis*-regulators for meiotic recombination hotspots. Encouraged by the results of our simulation study, we performed a GWAS analysis on the real HapMap data. For each pair of phenotype (i.e. a recombination hotspot) and genotype (i.e. a SNP), we used unpaired t-test to get the p-value of association between the recombination strengths and the SNP. To view the association, we did log transformation for the p-values and plotted the result in Figure 5(a), where the gradient colour intensities and sizes of dots represent the strengths of association. From Figure 5(a), we can see that there are strong and prevalent signals of *cis-*regulation shown as the diagonal red line on the plot, compared with *trans*-regulation. It could be that SNPs located inside or proximal to a recombination hotspot may change the DNA sequence thereby affecting the binding affinities of *trans*-factors. With a threshold of 1e-7.3 for the p-value, we identified 15,920 significantly associated SNPs. Then we extracted the flanking DNA sequences of these significant SNPs, each with a length of 1 kb. Using the software of FIMO [31], we matched the aforementioned 13-mer motif CCNCCNTNNCCNC (the binding motif of *PRDM9*) to these sequences. 45,293 motif occurrences were found in these 15,920 sequences. The strong *cis-*regulatory signal provides new evidence to confirm the relation of this 13-mer motif with recombination hotspots previously reported [6,9]. However, for the sophisticated regulation with meiotic recombination, it is unlikely that there is only one motif controlling all the recombination hotspots. In fact, this 13-mer motif can only explain a part of human hotspots. To search for other potential motifs, we fed these DNA sequences to the DREME software [32] for discriminative DNA motif discovery. From the output motifs, we selected three top-ranking motifs shown in Figure 5(b). The first motif has 9,882 positive occurrences in the 15,920 sequences with an E-value of 1.8e-591. The second motif has an E-value of 2.9e-565 with 3,537 positive occurrences in the sequences. The third motif, which is quite similar to the second one but ends with GG rather than CC, has 3,140 positive occurrences in the sequences with an E-value of 1.3e-456.

From the GWAS, we have not found any single SNP that is significantly associated with hotspots from different chromosomes. The number of hotspots affected by each single SNP was quite small compared with the sample size of 5,200 hotspots. For the three SNPs, *rs1874165*, *rs16874441*, and *rs3805547*, located inside *Prdm9 *gene, we only find one significant association between the three SNPs and one hotspot on Chromosome 5 as shown in Figure 5(c). For a *trans*-regulator like *PRDM9*, it is expected to show association with many hotspots, but it seems not the case according to our analysis. A reason might be that our GWAS method does not have sufficient statistical power to capture the *trans*-regulatory signals, e.g. the association might have been eliminated due to low MAF values in our pre-processing. It might also be the case that the assumption of two-allele recombination strengths (i.e. hot vs. cold) does not always apply for *PRDM9*. In addition, as demonstrated in [33], the hotspot activity can be influenced by multiple loci including both *cis*- and *trans*-effects.

To see whether the hotspot-SNP association is robust across different resolutions of recombination rate, we combined the recombination strengths of hotspots in the same chromosomes into 22 chromosome-wise recombination phenotypes. With the genotype of all tag SNPs, we did a GWAS analysis for these 22 phenotypes. The Manhattan plot in 6 shows the top 120 SNPs with significant associations (p-value less than 1e-7.3). Interestingly, these SNPs are mostly located near the ends of the chromosomes, implicating there might be an enrichment of regulators of genome stability at telomere regions of chromosomes. From the flanking DNA sequences of these 120 significant SNPs, two motifs were found using DREME and they turned out to be the same as the motif 1 and motif 2 found earlier by GWAS of individual hotspots, as shown in Figure 5(b).

**Figure 6:**
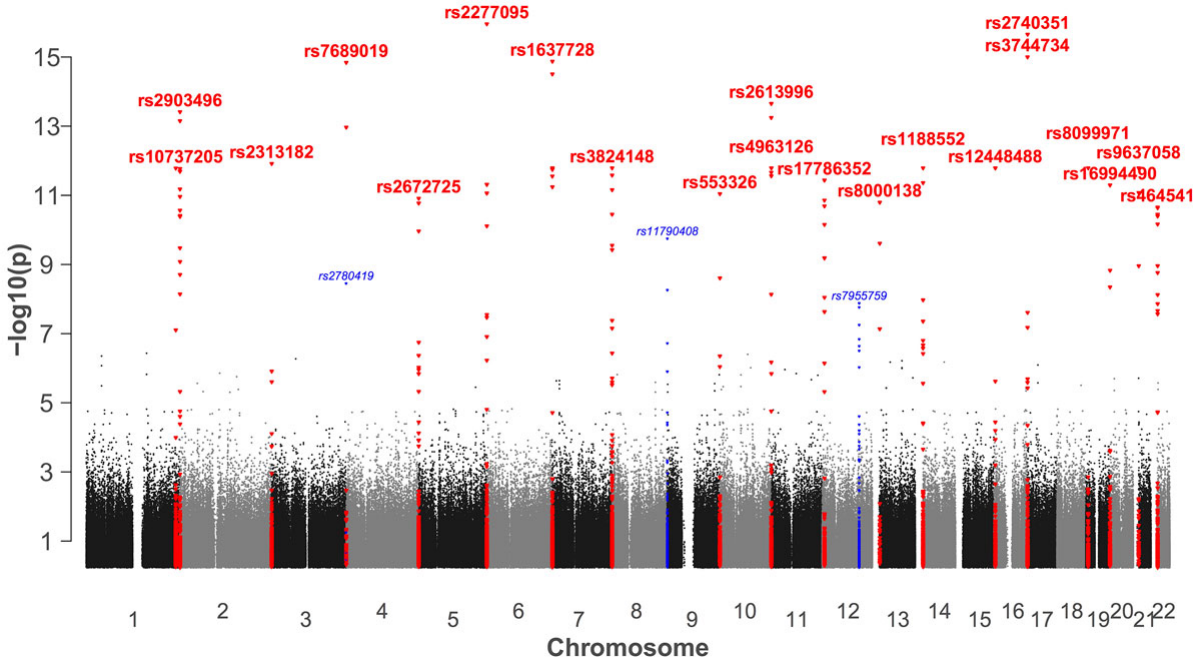
**Manhattan Plot of GWAS of chromosomal recombination phenotype**. Manhattan plot of chromosomal GWAS result is shown, where SNPs with p-values between 1e-10 and 1e-7.3 are labelled in blue, and SNPs with p-value less than 1e-10 are labelled in red.

### MHC screening

Recombination in the MHC (major histocompatibility complex) region on human chromosome 6 is of particular interest due to the roles of recombination in the generation of genetic diversity at HLA (Human Leukocyte Antigen) loci and the functions of MHC genes in the human immune system. Many studies have shown some associations between susceptibility to autoimmune diseases and particular alleles of MHC genes [34]. However the functional relationship between recombination and polymorphism of genes in MHC is not clear yet. Checking the GWAS results of hotspots in the MHC region, we found 10 hotspots significantly associated with 105 SNPs (p-value < 5e-8) (Additional File 3). All of these significantly associated SNPs are located inside the MHC region, except for two SNPs: *rs576205 *on chromosome 12, and *rs2061915 *on chromosome 19. Interestingly, the *rs2061915 *SNP is located inside the gene of *ZNF708 *which is one of the zinc finger genes reported to be expressed in human T cells [35,36]. And *rs576205 *is located in the gene of *KSR2 *which is a marker of immortalization, and was predicted to have a role in cell proliferation [37]. *KSR2 *also has association with the IL-2 expression through *mir-31 *thereby affecting the T cell differentiation [38]. These findings suggest that both *ZNF70*8 and *KSR2 *proteins may participate in the regulation of MHC activation or expression through the regulation of recombination in that region, which however needs to be verified through wet-lab experiments. Overall, our GWAS on MHC suggests that using ARG-walker is promising to help search for *trans-*regulators through GWAS.

## Conclusions

In this paper, we proposed a method named ARG-walker which is a graph mining algorithm for estimating individual-specific recombination strengths by random walks on ancestral recombination graphs. Most existing LD-based algorithms can only estimate the average recombination rate of a population. To the best of our knowledge, ARG-walker is the first computational method for estimating individual-specific strengths of recombination hotspots using only sequence polymorphism data. In most testing cases, ARG-walker performed well. In our simulation, ARG-walker can not only differentiate the recombination alleles of individuals, but also detect the cases of no variance in recombination frequency among individuals. Applying ARG-walker to the haplotype data of JPT+CHB population of HapMap Phase 3, we detected strong *cis*-regulatory signals that can corroborate the function of the 13-mer *PRDM9 *binding motif CCNCCNTNNCCNC, which is known to be critical for the regulation of meiotic recombination hotspots of human. In GWAS at both levels of hotspots and chromosomes, we identified two new motifs in the flanking regions of the significantly associated SNPs, i.e. AAAATANA and CNGCCTCC, which could be *cis*-regulators for the meiotic recombination hotspots of the human genome. Moreover, the significantly associated SNPs we detected in the combined chromosomal association study are mostly located near the ends of the chromosomes. It would be interesting to investigate the functional and evolutionary implication of this observation. By screening the GWAS results on the MHC region, two *trans-*regulatory SNPs were detected, and the genes flanking these two SNPs are both functionally related with the T-cell activation, demonstrating the potential of ARG-walker for the detection of *trans-*regulators of recombination hotspots. Nonetheless, ARG-walker and GWAS of recombination hotspots could be further improved by incorporating more advanced algorithmic and statistical techniques and new types of data (e.g. single-cell whole-genome sequencing data). Moreover, it would be interesting to also apply ARG-walker to understanding the relation of recombination hotspots with human autoimmune diseases.

## List of abbreviations used

ARG - ancestral recombination graph; LD - linkage disequilibrium; GWAS - genome-wide association study; BGC - biased gene conversion; MHC - major histocompatibility complex; MFA - minor allele frequency.

## Competing interests

The authors declare that they have no competing interests.

## Authors' contributions

JZ, TMP and PY envisioned the idea behind the ARG-walker method. CH and PY implemented the ARG-walker in Perl script and conducted simulation analysis and GWAS. HC and JZ did the analysis of the GWAS results. HC and JG did screening analysis on the MHC region. HC drafted the manuscript with input from JZ, PY, CKK and TMP. All authors read and approved the final manuscript.

## Supplementary Material

Additional file 1 **Python and Perl scripts for generating simulation data**. Python scripts for generating simulated SNP data, and Perl scripts for pre-processing the SNP data as input of ARG-walker. Click here for file

Additional file 2 **ARG-walker source code**. Perl scripts for the implementation of ARG-walker and a sample input file. Click here for file

Additional file 3 **Perl script for GWAS and supplementary MHC screening result**. A Perl script for the implementation of GWAS, and SNPs significantly associated with hotspots in the human MHC region. Click here for file

## Declaration

Publication charges for this article were funded by the Ministry of Education, Singapore.

This article has been published as part of *BMC Genomics *Volume 16 Supplement 12, 2015: Joint 26th Genome Informatics Workshop and 14th International Conference on Bioinformatics: Genomics. The full contents of the supplement are available online at http://www.biomedcentral.com/bmcgenomics/supplements/16/S12.
